# Phytochemical Analysis of Anti-Inflammatory and Antioxidant Effects of *Mahonia aquifolium* Flower and Fruit Extracts

**DOI:** 10.1155/2018/2879793

**Published:** 2018-06-27

**Authors:** Andra-Diana Andreicut, Alina Elena Pârvu, Augustin Cătălin Mot, Marcel Pârvu, Eva Fischer Fodor, Adriana Florinela Cătoi, Vasile Feldrihan, Mihai Cecan, Alexandru Irimie

**Affiliations:** ^1^Department of Pathophysiology, Faculty of Medicine, “Iuliu Hațieganu” University of Medicine and Pharmacy, 3-4 Victor Babes Street, RO-400012 Cluj-Napoca, Romania; ^2^Department of Chemistry, Faculty of Chemistry and Chemical Engineering, “Babes-Bolyai” University, 11 Arany Janos Street, RO-400028 Cluj-Napoca, Romania; ^3^Department of Biology, Faculty of Biology and Geology, “Babes-Bolyai” University, 42 Republicii Street, RO-400015 Cluj-Napoca, Romania; ^4^Medfuture Research Center for Advanced Medicine, “Iuliu Hațieganu” University of Medicine and Pharmacy, RO-400012 Cluj-Napoca, Romania; ^5^Institute of Oncology “I. Chiricuta”, 34-36 Republicii Street, RO-400015 Cluj-Napoca, Romania; ^6^Department of Immunology and Alergology, Faculty of Medicine, “Iuliu Hațieganu” University of Medicine and Pharmacy, 19-21 Croitorilor Street, RO-400162 Cluj-Napoca, Romania; ^7^Faculty of Medicine, “Iuliu Hațieganu” University of Medicine and Pharmacy, 8 Babes Street, RO-400012 Cluj-Napoca, Romania; ^8^Department of Oncology, Faculty of Medicine, “Iuliu Hațieganu” University of Medicine and Pharmacy, 34-36 Republicii Street, RO-400015 Cluj-Napoca, Romania

## Abstract

Oxidative stress and inflammation are interlinked processes. The aim of the study was to perform a phytochemical analysis and to evaluate the antioxidant and anti-inflammatory activities of ethanolic *Mahonia aquifolium* flower (MF), green fruit (MGF), and ripe fruit (MRF) extracts. Plant extract chemical composition was evaluated by HLPC. A DPPH test was used for the *in vitro* antioxidant activity. The *in vivo* antioxidant effects and the anti-inflammatory potential were tested on a rat turpentine oil-induced inflammation, by measuring serum nitric oxide (NOx) and TNF-alpha, total oxidative status (TOS), total antioxidant reactivity (TAR), oxidative stress index (OSI), 3-nitrothyrosine (3NT), malondialdehyde (MDA), and total thiols (SH). Extracts were administrated orally in three dilutions (100%, 50%, and 25%) for seven days prior to inflammation. The effects were compared to diclofenac. The HPLC polyphenol and alkaloid analysis revealed chlorogenic acid as the most abundant compound. All extracts had a good *in vitro* antioxidant activity, decreased NOx, TOS, and 3NT, and increased SH. TNF-alpha was reduced, and TAR increased only by MF and MGF. MDA was not influenced. Our findings suggest that *M. aquifolium* has anti-inflammatory and antioxidant effects that support the use in primary prevention of the inflammatory processes.

## 1. Introduction

The relation between antioxidants and degenerative diseases is a topic that focuses the attention of many researchers nowadays [1]. Reactive oxygen species (ROS) result from the oxidative processes in every living organism, as part of the aerobic metabolism. They are represented by superoxide anion, hydrogen peroxide, and hydroxyl radicals [2]. In small doses, they are useful and play physiological roles and are also involved in signalling processes [3]. When the antioxidant system is overloaded, ROS will damage proteins, DNA, and lipids [4]. Therefore, it is essential to identify exogenous sources of antioxidants which can reduce ROS effects [5].

Plants represent an important source of protective agents, due to their content of polyphenols, vitamins, fiber, phytosterols, and carotenoids [6]. Polyphenols have both antioxidant and prooxidant properties. The antioxidant activity is due to the scavenging effect of free radicals [7] and ensures the protection of intracellular structures against oxidative stress, favouring cell viability [8].

As prooxidants, polyphenols may stimulate apoptosis and inhibit tumour growth [8]. Polyphenols have good effects on degenerative diseases like cancer, cardiovascular diseases, diabetes, and osteoporosis [9]. As for their effect on the cardiovascular system, polyphenols reduce blood pressure, inflammation, and oxidative markers, they prevent endothelial dysfunction [10], they are antithrombotic, and they act as vasodilators [11]. They also inhibit the proinflammatory activity of cyclooxygenase (COX), lipooxygenase (LOX), and inducible nitric oxide synthase (iNOS) [12]. As protectors for the endothelial function, polyphenols act in the early stages of the atherosclerotic process by reducing LDL oxidation [12].

Genus *Mahonia* is the second largest one from the *Berberidaceae* family. The plants from this genus were used in traditional medicine as a treatment for psoriasis, dermatitis, fungal infections, tuberculosis, dizentheria, and wounds [13]. From all *Mahonia* species, *Mahonia aquifolium* is the most cultivated in Turkey [14]. Due to its high content in alkaloids, *M. aquifolium* has antioxidant, anti-inflammatory, [15, 16], hypoglycemic, hepatoprotective, and hypotensive properties [17]. In the cardiovascular system, *M. aquifolium* alkaloids induce vasodilatation by blocking *Ca*^2+^ entrance in the cells [18] and act as alpha-1 adrenoreceptor antagonists [19].

A variety of fruits are known for having anti-inflammatory and vasodilatation properties [5]. Fruits from *M. aquifolium* are light yellow and bloom in April, but less information is known about their effects [14]. However, the fruits from a *Mahonia* were used in the treatment of insomnia, tinnitus, and dizziness [20].

Considering all these previous findings, the present work aimed at performing a phytochemical analysis and investigating the antioxidant and anti-inflammatory activity of the ethanolic *M. aquifolium* flower and fruit extracts.

## 2. Materials and Methods

### 2.1. Plant Material

Fresh *Mahonia aquifolium* (Pursh) Nutt. flowers and fruits were purchased from the A. Borza Botanical Garden “Babes-Bolyai” University of Cluj-Napoca, Romania between April and June 2015 and extracted in the Mycology Laboratory of “Babes-Bolyai” University, Cluj-Napoca, Romania, by a modified Squibb repercolation method with 70% ethanol (Merck, Bucuresti, Romania), producing the following extracts of *M. aquifolium*: green fruit extract 1 : 1 (g : mL) (MGF), ripe fruit extract 1 : 1 (g : mL) (MRF), and flower extract 1 : 1 (g : mL) (MF) [21]. The plants were taxonomically identified and authenticated, and voucher specimens (number 665978) were deposited in the Herbarium of “A. Borza” Botanical Garden, “Babes-Bolyai” University of Cluj-Napoca, Romania.

### 2.2. Phytochemical Analysis

A HPLC-DAD approach was used to separate and quantitatively determine the polyphenols and alkaloids. In the first chromatographic approach, the assays were performed on an Agilent 1200 HPLC system (Waldbronn, Germany) equipped with an online vacuum degasser, quaternary pump, temperature-controlled sample tray, automatic injector, a column thermostat compartment, and a DAD detector. The chromatographic separations were run on a Nucleosil 100 C_18_ column (240 mm × 4.6 mm, 5 *μ*m particle size) from Macherey-Nagel (Duren, Germany). The injection volume was 5 *μ*L (0.2 *μ*m filtered extract), the column temperature was set at 25°C, and the flow rate was 1.2 mL/min. Several preliminary tests were employed for method optimization by varying the experimental conditions. The optimum method consisted of a gradient elution using solvent A, 10 mM of ammonium acetate pH 5, and solvent B as acetonitrile. The gradient was as follows: 0–15 min from 8 to 30% B, 15–25 min isocratic at 30% B, 25–35 min from 30% to 85% B, 35–38 min from 85% to 95% B, 38–39 min isocratic at 95% B and 39–39.1 min back to 8% B where it was kept until 40 min. As standards, there were chlorogenic acid, *p*-coumaric acid, ferulic acid, rutin, isoquercitrin, quercetin, berbamine, jatrorrhizine, palmatine, and berberine, all of analytical grade purity from different commercially available sources (Sigma-Aldrich, Germany). A calibration curve was constructed for each compound at 11, 22, 44, 88, 175, and 340 *μ*g/mL using the area of the peak by integration employed by the Agilent software. The limit of quantification (LOQ) and limit of detection (LOD) were determined by the formulas LOQ = (10 × standard deviation of intercept)/calibration curve slope and LOD = (3.3 × standard deviation of intercept)/calibration curve slope, respectively. The UV-Vis detection of the compounds has been accomplished using the DAD detector that measured the entire spectrum in the 210–700 nm region every 1 s, and the chromatograms were monitored at 220, 280, 340, and 425 nm. The identification of the compounds was employed by both chromatographic retention time (with a 0.3 s as tolerance) and spectral similarities (higher than 99.9% was considered as positive) which were done by the built-in software. The chromatograms were exported and the graphs were developed in Excel.

### 2.3. *In Vitro* Antioxidant Effects

The 1,1-diphenyl-2-picrylhydrazyl (DPPH) free radical scavenging assay was used for the evaluation of the antioxidant capacity of the investigated extracts. Briefly, in 3 mL of each diluted extract, a 1 mL DPPH and 0.1 mM methanol solution was added. Blanks were included replacing extract volumes for acetone/water. After 30 min in the dark and at room temperature, mixture absorbance was measured at 517 nm against a blank. The percentage of the radical scavenging activity of each extract was calculated using the following formula:

percentage of radical scavenging activity (AA%) = [(OD control − OD sample)/OD control] × 100. AA% was converted to Trolox equivalents using a calibration curve of Trolox standard solutions (0.5–5 *μ*g/mL). The concentration required to scavenge 50% of DPPH free radicals (IC50) was calculated [22].

### 2.4. Experimental Design

For the present study, 12 groups (*n* = 5) of male albino Wistar rats with body weights between 200 and 250 g were used. They were purchased from the Animal Facility of “Iuliu Hațieganu” University of Medicine and Pharmacy. The rats were kept in common polypropylene cages under controlled conditions (12 h light/dark cycles, at an average temperature of 21-22°C), with free access to a standard pellet diet (Cantacuzino Institute, Bucharest, Romania) and water *ad libitum*. Three ethanolic extracts of *M. aquifolium* were tested*:* ripe fruits (MRF), green fruits (MGF), and flowers (MF). For seven days, the mentioned extracts were administered orally by gavage (1 mL/animal) in three different dilutions, respectively: 100%, 50%, and 25%. Tap water (1 mL/animal) was administrated by gavage for seven days to the animals from the negative control group (CONTROL) and for the positive inflammation group (INFLAM). An anti-inflammatory control group was treated by gavage for seven days with diclofenac (10 mg/kg b.w.) (DICLO) [23]. Inflammation was induced with turpentine oil (6 mL/kg b.w.) administered intramuscularly, in the animals treated with the extracts, as well as in the INFLAM and DICLO groups [24]. To the CONTROL animals, 0.9% saline was injected intramuscularly (i.m.). One day after the inflammation induction, 60 mg/kg b.w. ketamine and 15 mg/kg b.w. xylazine were used to anesthetize the rats [25], blood was withdrawn by retroorbital puncture, and serum was stored at −80°C until use. The experiments were performed in triplicate. All the animals were used only once, and they were killed by cervical dislocation immediately after the assay.

### 2.5. The Anti-Inflammatory Effect Evaluation

To evaluate the anti-inflammatory effects of the ethanolic plant extracts, nitric oxide (NO) and TNF-alpha were measured.

For NO synthesis, the Griess reaction was used as an indirect method which measures total nitrites and nitrates (NOx) as previously described [26]. The concentration of serum NOx was expressed as nitrite *μ*mol/L [27].

Serum TNF-alpha was measured using a rat ELISA kit (MBS175904) that applies the quantitative sandwich enzyme immunoassay technique.

### 2.6. The Antioxidant Effect Evaluation

The total oxidative status (TOS) of the serum was measured using a colorimetric assay [26, 28]. The assay results are expressed in *μ*mol H2O2 equiv./L.

The total antioxidant response (TAR) was measured in serum using a colorimetric assay [26, 29]. The results are expressed as *μ*mol Trolox equiv./L.

The oxidative stress index (OSI) is the ratio of the TOS to the TAR and it is an indicator of the oxidative stress level [26, 30]: OSI (arbitrary unit) = TOS (*μ*mol H2O2 equiv./L)/TAR (*μ*mol Trolox equiv./L).

Peroxynitrite formation was assessed indirectly by measuring serum 3-nitrotyrosine (3NT) using a rat ELISA kit (MBS732683) that applies the quantitative sandwich enzyme immunoassay technique.

Malondialdehyde (MDA) was assessed using thiobarbituric acid, as previously described [26, 31]. Serum MDA concentration was expressed as nmol/mL of serum.

Total thiols (SH) were measured using Ellman's reagent [26, 32]. Serum SH concentration was expressed as mmol GSH/mL.

All of the spectroscopic measurements were performed using a Jasco V-530 UV-Vis spectrophotometer (Jasco International Co. Ltd., Tokyo, Japan).

### 2.7. Statistical Analysis

Data are expressed as mean ± SD, averaged over at least three independent experiments for normally distributed data. Otherwise, the median, first quartile (Q1), and third quartile (Q3) were reported. Comparisons among groups, in all studied parameters, were analyzed by using the one-way analysis of variance (ANOVA) test and Bonferroni-Holm post hoc test. *p* < 0.05 was considered statistically significant. Correlations among data obtained were calculated using Pearson's correlation coefficient (*r*). All analyses were performed using the program “Statistical Package for Social Sciences (SPSS) version 16” (SPSS Inc., Chicago, IL, USA).

## 3. Results

### 3.1. Phytochemical Analysis

**Figure 1:**
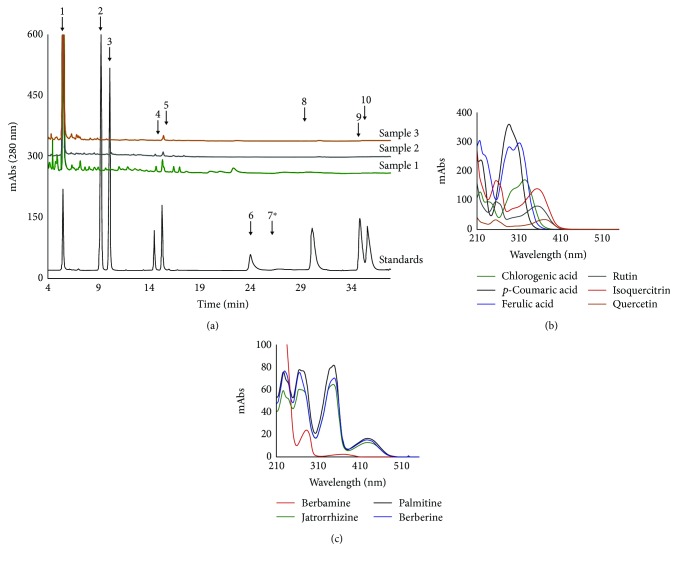
(a) Chromatograms at 280 nm of the *M. aquifolium* flower, green fruit, and ripe fruit extracts. The ten standards are indicated by arrows and numbers. Berbamine (7^∗^) is barely visible in the 280 nm chromatogram, but it is much better detected and quantified separately from the 220 nm chromatogram. (b) HPLC-DAD registered absorption molecular spectra in the UV-vis domain for the polyphenolic standards at 350 *μ*g/mL. (c) HPLC-DAD registered absorption molecular spectra in the UV-vis domain for the alkaloid standards at 350 *μ*g/mL. 1—Chlorogenic acid, 2—*p*-coumaric acid, 3—ferulic acid, 4—rutin, 5—isoquercitrin, 6—quercetin, 7—berbamine, 8—jatrorrhizine, 9—palmatine, and 10—berberine. Sample 1—*Mahonia aquifolium* flowers, sample 2—*Mahonia aquifolium* ripe fruits, and sample 3—*Mahonia aquifolium* green fruits.

In the present study, we measured six polyphenols (chlorogenic acid, *p*-coumaric acid, ferulic acid, rutin, isoquercitrin, and quercetin) and four alkaloids (berbamine, jatrorrhizine, palmatine, and berberine) (Table 1, Figure 1). We identified and quantified chlorogenic acid, ferulic acid, and *p*-coumaric acid as hydroxycinnamic acid derivates (Table 1). Chlorogenic acid was found in the highest concentration in MF (2013 ± 2 *μ*g/mL), followed by MGF (1763 ± 7 *μ*g/mL) and MRF (944 ± 22 *μ*g/mL). We could quantify *p*-coumaric acid only in MF (7.2 ± 0.1 *μ*g/mL) and MRF (4.5 ± 2.0 *μ*g/mL). MF was richer in *p*-coumaric acid (10.0 ± 0.3 *μ*g/mL) than MRF (7.8 ± 0.0 *μ*g/mL) and MGF (5.6 ± 0.2 *μ*g/mL) were. In all ethanolic extracts, we identified two flavonoid glycosides, namely rutin and isoquercitrin. Rutin was more abundant in MF (73 ± 1.6 *μ*g/mL) than in MRF (26.1 ± 0.0 *μ*g/mL) and MGF (12.9 ± 0.0 *μ*g/mL). Isoquercitrin was higher in MGF (37.3 ± 0.2 *μ*g/mL) than in MF (29.7 ± 0.7 *μ*g/mL) and MRF (32.4 ± 0.3 *μ*g/mL). In MF, MGF, and MRF, the tested alkaloids were in concentrations under LOD.

### 3.2. *In Vitro* Antioxidant Activity

The ethanolic extracts of *M. aquifolium* had a good DPPH radical scavenging activity (Table 2). Trolox IC50 was 11.2 *μ*g/mL. Considering that an antioxidant activity with an IC50 between 50 and 100 *μ*g/mL is good and one that has between 100 and 200 *μ*g/mL is weak, MF IC50 (60.82 *μ*g/mL) and MGF IC 50 (81.6 *μ*g/mL) had a good antioxidant activity and MRF IC50 (135.74 *μ*g/mL) has a weak antioxidant activity.

### 3.3. *In Vivo* Anti-Inflammatory and Antioxidant Effects

TNF-alpha was increased in the inflammation group (*p* < 0.01). TNF-alpha was reduced exclusively by MGF50 and MF25 (*p* < 0.05) and no significant effect was found for the rest of the tested extracts (*p* > 0.05) (Table 3).

NOx was significantly increased in the inflammation group (*p* < 0.01) and an important reduction was found in the group where diclofenac was administered (*p* < 0.01). Compared to the inflammation group, MRF extracts reduced NOx significantly (*p* < 0.01), MRF100 being the most efficient. MRF effects were comparable to that of diclofenac (*p* > 0.05). There was no significant inhibitory activity on NOx in all MF dilutions (*p* > 0.05). From the MGF samples, only MGF50 had a small inhibitory effect on NOx (*p* < 0.05) (Table 3).

TOS analysis showed that inflammation caused an important increase (*p* < 0.01) and diclofenac caused a significant reduction (*p* < 0.001). All MGF extracts reduced TOS (*p* < 0.01), but not as much as diclofenac (*p* > 0.05). MRF extracts did not influence TOS significantly (*p* > 0.05). MF reduced TOS, but only MF100 had a significant inhibitory effect (*p* < 0.01).

TAR was reduced in the inflammation group (*p* < 0.05), and it was slightly increased when diclofenac was administered (*p* < 0.05). MGF increased TAR, MGF25 being the better stimulator (*p* < 0.001). The MGF effect was as good as that of diclofenac (*p* > 0.05). MF100 and MF50 increased TAR (*p* < 0.05), but this effect was smaller than diclofenac (*p* < 0.001). MRF extracts had no significant effects on TAR (*p* > 0.05) (Table 4).

OSI was increased in the inflammation group (*p* < 0.01) and decreased when diclofenac was administrated (*p* < 001). Only MF100 extracts decreased OSI (*p* < 0.05), but the effect was smaller than that of diclofenac (*p* < 0.05). MGF extracts were good inhibitors of OSI (*p* < 0.01), but the effect was smaller than that of diclofenac (*p* > 0.05). The MRF extract had no significant effect on OSI (*p* > 0.05) (Table 4).

3NT was increased in the inflammation group (*p* < 0.01) and was reduced significantly after diclofenac (*p* < 0.001). MGF were also good inhibitors of 3NT, MGF100 being more efficient (*p* < 0.001) than MGF50 (*p* < 0.01) and MGF25 (*p* < 0.05). From the MRF, only MRF25 reduced 3NT (*p* < 0.001). MF extracts decreased 3NT significantly (*p* < 0.01) (Table 4). MDA production was significantly increased in the inflammation group (*p* < 0.001), and diclofenac treatment caused an important decrease of MDA (*p* < 0.001). Only MRF100 (*p* < 0.01) and MRF50 (*p* < 0.05) reduced MDA. MF and MGF did not influence significantly the production of MDA (*p* > 0.05) (Table 4).

Inflammation caused a reduction of SH (*p* < 0.001), and diclofenac caused an important increase (*p* < 0.01). SH was increased by all *M. aquifolium* extracts (*p* < 0.01–0.001), and the effects were better than the effect of diclofenac (Table 4).

## 4. Discussion

In the present study, a phytochemical analysis of the ethanolic *M. aquifolium* flower, green fruit, and ripe fruit extracts, was performed for the first time. The antioxidant activity and anti-inflammatory effects were proved.

From the three hydroxycinnamic acid derivates measured in the ethanolic *M. aquifolium* extracts, the main component was chlorogenic acid. MF had a higher content of chlorogenic acid than MGF and MRF. This was an important result because chlorogenic acid improves the risk of cardiovascular and associated diseases by reducing the levels of free fatty acids and triglycerides [33]. Due to its antioxidant properties, chlorogenic acid also has a protective role in cardiovascular diseases by increasing NO production [34]. In hypertension, it has a vasodilator effect and reduces ROS production [35]. Chlorogenic acid also inhibits platelet aggregation and reduces blood viscosity [36].

Ferulic acid was found in much lesser concentrations. It was higher in MF than in MRF and MGF. Ferulic acid has antioxidant properties [37, 38] by scavenging ROS and activating DNA repair [39]. It interferes with insulin, ghrelin, and leptin to prevent weight gain and the accumulation of intra-abdominal fat [40]. Ferulic acid also has anti-inflammatory, anticancer, and cardioprotective effects [41]. Due to its antioxidant properties, it is beneficial in epilepsy [42] and other chronic neurological conditions [43]. It is also an antidepressant [44], and it improves memory [45].

The *p*-coumaric acid was found only in MRF and MF. It is a phenolic acid with antioxidant [46], anti-inflammatory, cardioprotective [47], hepatoprotective, and nefroprotective properties [48]. It reduces the lipoprotein peroxidation process and reduces the free radicals [46, 49]. The *p*-coumaric acid has anticancer activity by inducing apoptosis and cell cycle arrest [48].

From the three measured flavonoid glycosides, only rutin and isoquercitrin were found in MF, MGF, and MRF. Quercetin was under the LOD.

Rutin is a flavonoid with antioxidant, anti-inflammatory, antidiabetic, and antiobesity properties [50, 51]. Due to its antioxidant capacity, rutin has cardioprotective effects, reducing LDH, CK-MB, ROS, and apoptosis [52, 53]. Rutin protects against endothelial damage and reduces the occurrence of chronic complications in type 2 diabetes [42, 54]. The higher level of rutin concentration in MF was correlated with a good DPPH scavenging activity. Isoquercitrin is a glycated flavonoid [55] with a strong antioxidant activity due to its capacity to scavenge free radicals [56] and to inhibit arginase, favouring NO production [57]. This phenolic compound has anticancer [58] and antiapoptotic effects [59].

The isoquinoline alkaloids are the major subclass of alkaloids of the genus *Mahonia* [26]. The previously identified alkaloids belong to three major classes: protoberberines, aporphines, and bisbenzylisoquinolines. Berberine is the most widely distributed alkaloid in the *Mahonia* species [60], but other protoberberines, including palmatine, jatrorrhizine, berbamine, columbamine, and coptisine were also found in these species [20]. The analysis of *M. aquifolium* extracts looked for the presence of berberine, palmatine, jatrorrhizine, and berbamine but due to the trace amounts of these alkaloids they were under LOD.

The phytochemical analyses suggested the possible anti-inflammatory and antioxidant effects of the *M. aquifolium* fruit and flower extracts.

The *in vitro* antioxidant activity was assessed with the DPPH test. MF and MGF proved to have a good antioxidant effect, but MRF had just a weak antioxidant activity. For MF, DPPH correlated with the high chlorogenic acid, feluric acid, and rutin content and for MGF with the high chlorogenic acid and isoquercitrin content.

Furthermore, the present study evaluated the *in vivo* anti-inflammatory and antioxidant effects in an experimental rat acute inflammation induced by turpentine oil, a nonantigenic inflammatory stimulus [61] that activates inflammatory cytokines and NO release. High serum levels of TNF alpha and NOx were positive markers of the inflammatory response. Only MF and MGF proved to have important anti-inflammatory effects by reducing TNF alpha.

NO is the product with the smallest molecular mass secreted in the mammalian cells. It has a high chemical reactivity and a short lifetime and specificity [62]. NO is generated by 3 isoforms of NOS, respectively, inducible NOS (iNOS), neuronal NOS (nNOS), and endothelial NOS (eNOS) [63]. The nNOS and eNOS are expressed constitutively and produce small quantities of NO. The iNOS is expressed after immunological and inflammatory stimuli and acts like a protector agent [64]. Low levels of NO induce normal physiological signalling, leading to antioxidant reactions. In intermediate concentrations, NO stimulates anti-inflammatory and immunosuppressive responses, has antiapoptotic, progrowth, and angiogenic effects. High NO levels have antiproliferative effects and induce cell cycle delay. Moreover, if there is a prolonged NO increase, it may induce apoptosis [65]. When iNOS is synthesised in high quantities, NO also reacts fast with superoxide anion and will form peroxynitrite (ONOO−), a nonradical reactive species responsible for most of the NO pathological effects [66–68]. Previous studies suggest that plants play an important anti-inflammatory role, because they inhibit NO synthesis [24]. In the present study, only MRF100 proved to have important anti-inflammatory effects by reducing NO synthesis and it was comparable with that induced by diclofenac. Treatment with MGF had a weak inhibitory effect, and MF had no important effect upon NOx. Because the MRF effect on NOx was not correlated with the *in vitro* DPPH test, it may be presumed that the antioxidant activity was not significantly involved. In some human diseases, antioxidant therapy failure was called the antioxidant paradox [69]. Due to the fact that overproduction of ROS can induce an inflammatory response, and inflammatory mediators can induce an oxidative stress, it was generally accepted that oxidation and inflammation are interlinked processes [70]. The latest explanation of antioxidant therapy failure comes from the finding that antioxidants do not inhibit oxidative stress and the associated inflammation at the same time [71].

Oxidative stress is destructive and it represents the dysbalance between antioxidants and oxidants, in favour of the last ones [72, 73]. The most important oxidants are the reactive oxygen species (ROS), which include superoxide anion, hydrogen peroxide, and hydroxyl radicals [2]. In small to moderate concentrations, ROS have physiologic roles [3], acting as signalling molecules, being involved in cell growth, intercellular adhesion, cellular differentiation, and apoptosis [73, 74]. In high concentrations, ROS are highly reactive molecules that may damage proteins, lipids, and DNA [2, 3, 75] and favour atherosclerosis, cancer, and ageing [76]. The measurement of the stable markers in circulation during oxidative stress [77–79] is a helpful way to appreciate plant extract effects. The oxidative stress biomarkers may be classified as molecules that are modified by interactions with ROS (e.g., DNA, lipids, proteins and carbohydrates) and molecules of the antioxidant system that change in response to increased redox stress [77]. For total oxidative status (TOS) evaluation, diverse methods were developed [28]. In our study, serum TOS was higher in the inflammation group. MF and MGF lowered serum TOS more than MRF. These results positively correlated with the DPPH test.

The antioxidant mechanisms are given in terms of the capacity to scavenge free radicals, to chelate metals, and to act in a synergic manner with other antioxidants. There are two groups of methods used for the determination of the total antioxidant capacity: those based on single-electron transfer monitored spectrophotometrically by a color change due to the free radical reduction, and those based on hydrogen atom transfer measured by the elimination of peroxyl radicals [80]. The antioxidant capacity of the plasma is represented by the thiol groups of proteins and uric acid [81]. Serum TAR [82] was lower in the inflammation group, and only the treatments with MF and MGF extracts increased TAR. In these extracts, we found higher levels of chlorogenic acid, rutin, and isoquercitrin, compounds known to have antioxidant activities.

OSI assesses the global oxidant/antioxidant balance in the living organisms [83]. It was decreased by the MGF and MF treatments, and this was correlated positively with TOS and negatively to TAR.

3NT is a product of protein tyrosine nitration mediated by peroxynitrite anion and nitrogen dioxide. Therefore, it is a good marker of oxidative cell injury and inflammation, as well as NO production [84]. Increased oxidative stress increases ROS and 3NT and consumes NO. Increased levels of 3NT were associated with atherosclerosis [85] and observed in patients with coronary dysfunction, as well as after the removal of the cardiovascular risk factors and normalization of C reactive protein [77]. All tested *M. aquifolium* extracts reduced 3NT, but MF and MGF were more effective than MRF. The inhibitory effect on 3NT was correlated positively with TOS reduction and negatively with TAR elevation. These results could be explained by the better MF and MGF *in vitro* DPPH tests.

Malondialdehyde (MDA) results from arachidonic acid, a polyunsaturated fatty acid, and it is a secondary product of lipid peroxidation [86]. Aldehydes are toxic because they interact with DNA and proteins favouring mutations, which are risk factors for cancer, atherosclerosis, and other cardiovascular diseases [87]. The link between MDA and atherosclerosis is its reaction with lipoproteins and the formation of arterial foam cells [88, 89]. Collagen has a high reactivity for MDA, and this will increase heart and vessel rigidity [87]. High levels of MDA were correlated also with myocardial infarction [90], cardiovascular complications of haemodialysis, diabetes, preeclampsia [87], congestive heart failure, and with the severity of the disease [91]. Only treatment with MRF decreased MDA, and it was negatively correlated with TAR.

Protein thiol groups are important determinants of the total antioxidant capacity. They contain a sulfhydryl group which can transform into disulfide bonds when oxidized by the oxygen molecules. This reaction is reversible, so the disulfide bonds can turn back into thiols [92]. The chemical versatility allows them to participate in different processes like signalling, antioxidant defence, and structural stabilization [93]. Studies show that albumin, the most thiol-abundant protein, is implied in the reduction of blood pressure and cardiovascular risk [94]. All tested *M. aquifolium* ethanolic extracts increased serum SH, proving that these extracts may improve the antioxidant defence.

## 5. Conclusion

Considering the study results, we concluded that *M. aquifolium* flower, green fruit, and ripe fruit ethanol extracts have good *in vitro* and *in vivo* antioxidant activities, and good anti-inflammatory effects. The efficiency varies with plant organ phytochemical composition. *M. aquifolium* flower, green fruit, and ripe fruit extracts may be considered for therapeutic interventions needing simultaneous antioxidant and anti-inflammatory effects.

## Figures and Tables

**Table 1:** HPLC analysis of the M. aquifolium flower, green fruit, and ripe fruit extracts.

Number	Compounds	*t*_elution_ (min)	_*R*_2	LOD (*μ*g/mL)	LOQ (*μ*g/mL)	Sample1 MF (*μ*g/mL)	Sample2 MRF (*μ*g/mL)	Sample3 MGF (*μ*g/mL)
1	Chlorogenic ac.	5.41	0.9991	3.2	9.8	2013 ± 2	944 ± 22	1763 ± 7
2	*p*-Coumaric ac.	9.17	0.9999	1.3	4.0	7.2 ± 0.1	4.5 ± 2.0	<LOQ
3	Ferulic ac.	10.07	0.9998	1.4	4.2	10.0 ± 0.3	7.8 ± 0.	5.6 ± 0.2
4	Rutin	14.55	0.9996	2.7	8.1	73 ± 1.6	26.1 ± 0.0	12.9 ± 0.0
5	Isoquercitrin	15.34	0.9995	1.7	5.2	29.7 ± 0.7	32.4 ± 0.3	37.3 ± 0.2
6	Quercetin	24.1	0.9949	13.7	41.6	<LOD	<LOD	<LOD
7	Berbamine	25.5	0.9997	2.4	7.3	<LOD	<LOD	<LOD
8	Jatrorrhizine	30.57	0.9994	2.7	8.3	<LOD	<LOD	<LOD
9	Palmatine	34.96	0.9998	1.7	5.2	<LOD	<LOD	<LOD
10	Berberine	36.08	0.9996	2.1	6.4	<LOD	<LOD	<LOD

MF—*M. aquifolium* flowers, MGF—*M. aquifolium* green fruits, MRF—*M. aquifolium* ripe fruits, LOD—limit of detection, LOQ—limit of quantification, and *R*^2^—coefficient of determination for the calibration curves (at six levels of concentrations). Indicated intervals represent the average ± standard deviation (*n* = 3).

**Table 2:** *In vitro* DPPH radical scavenging activity of *M. aquifolium* flower, green fruit, and ripe fruit extracts.

MGF		MRF		MF	
(*μ*g/mL)	(AA%)	(*μ*g/mL)	(AA%)	(*μ*g/mL)	(AA%)
750	94.6	1000	83.1	375	88
562.5	86.65	750	73.13	250	79.95
375	76.65	500	67.82	125	65.6
187.5	65.17	250	61		

MGF—*M. aquifolium* green fruits, MRF—*M. aquifolium* ripe fruits, MF—*M. aquifolium* flowers, and AA%—percentage of radical scavenging activity.

**Table 3:** *In vivo* anti-inflammatory effects of *M. aquifolium* flower, green fruit, and ripe fruit extracts.

	NOx	(*μ*mol/L)	TNF	(pg/mL)
CONTROL	48,489 ± 12,975		116,634 ± 0.678	
INFLAM	75,234 ± 12,136^∗∗^		140,594 ± 15,960^∗∗∗^	
DICLO	52,318 ± 5389^∗∗^		127,228 ± 10,332	
MGF100%	72,406 ± 9292^∗^		143,564 ± 16,419	
MGF50%	57,473 ± 9110^∗^		118,317 ± 13,854^∗^	
MGF25%	62,686 ± 10,155^∗^		133,168 ± 15,522	
MRF100%	46,898 ± 6766^∗∗∗^		130,941 ± 17,836	
MRF50%	53,172 ± 3981^∗∗^		146,287 ± 15,092	
MRF25%	53,526 ± 7582^∗∗^		121,782 ± 15,522	
MF100%	59,623 ± 10,224^∗^		132,178 ± 21,138	
MF50%	69,638 ± 7507		129,951 ± 16,419	
MF25%	62,421 ± 7539		115,842 ± 10,632^∗^	

3NT—3-nitrithyrosine; *M. aquifolium*: MGF—green fruits, MRF—ripe fruits, and MF—flowers; ^∗^*p* < 0.05, ^∗∗^*p* < 0.01, and ^∗∗∗^*p* < 0.001.

**Table 4:** *In vivo* antioxidant effects of *M. aquifolium* flower, green fruit, and ripe fruit extracts.

	(*μ*mol H_2_O_2_ TOS equiv./L)	(mmol TA Trolox R equiv./L)	OSI	3NT	MD (nmol A MDA/L)	(mmol SH GSH/L)
CONTROL	29.58 ± 1.85	1.09 ± 0.001	27.13 ± 1.67	0.34 ± 0.06	4.31 ± 0.89	0.67 ± 0.09
INFLAM	41.58 ± 6.55^∗∗^	1.08 ± 0.0007^∗^	38.20 ± 6.009^∗∗^	0.67 ± 0.18^∗∗^	7.62 ± 0.62^∗∗∗^	0.42 ± 0.06^∗∗^
DICLO	24.92 ± 3.02^∗∗∗^	1.08 ± 0.0004^∗^	22.87 ± 2.77^∗∗∗^	0.29 ± 0.02^∗∗∗^	5.49 ± 0.72^∗∗∗^	0.56 ± 0.05^∗∗^
MGF100%	26.19 ± 7.37^∗∗^	1.09 ± 0.0011^∗∗^	24.03 ± 6.74^∗∗^	0.29 ± 0.04^∗∗∗^	6.90 ± 0.47	0.66 ± 0.11^∗∗^
MGF50%	32.09 ± 5.63^∗^	1.09 ± 0.0006^∗∗^	29.45 ± 5.15^∗^	0.32 ± 0.02^∗∗^	6.97 ± 0.54	0.67 ± 0.10^∗∗^
MGF25%	30.60 ± 4.59^∗∗^	1.09 ± 0.0008^∗∗∗^	28.07 ± 4.19^∗∗^	0.38 ± 0.14^∗^	6.90 ± 0.47	0.59 ± 0.11^∗∗^
MRF100%	39.21 ± 5.31	1.08 ± 0.0008	36.02 ± 4.86	0.41 ± 0.27	6.00 ± 0.66^∗∗^	0.77 ± 0.14^∗∗^
MRF50%	34.49 ± 6.64	1.08 ± 0.0006	31.70 ± 6.10	0.42 ± 0.26	6.54 ± 0.86^∗^	0.83 ± 0.20^∗∗^
MRF25%	34.85 ± 7.85	1.08 ± 0.0003	32.03 ± 7.21	0.29 ± 0.03^∗∗∗^	7.73 ± 0.29	0.67 ± 0.14^∗∗^
MF100%	30.18 ± 4.74^∗∗^	1.08 ± 0.0003^∗^	27.76 ± 4.36^∗∗^	0.32 ± 0.06^∗∗^	7.15 ± 0.97	0.66 ± 0.10^∗∗^
MF50%	39.65 ± 4.51	1.08 ± 0.001	36.43 ± 4.12	0.45 ± 0.25	7.12 ± 0.82	0.77 ± 0.05^∗∗^
MF25%	32.79 ± 6.77	1.08 ± 0.001	30.12 ± 6.21	0.28 ± 0.03^∗∗∗^	7.68 ± 0.97	0.75 ± 0.14^∗∗^

TOS—total oxidative status, TAR—total antioxidant reactivity, OSI—oxidative stress index, 3NT—3-nitrithyrosine, MDA—malondialdehyde, SH—total thiols, MGF—*M. aquifolium* green fruits, MRF—*M. aquifolium* ripe fruits, and MF—*M. aquifolium* flowers; ^∗^*p* < 0.05, ^∗∗^*p* < 0.01, and ^∗∗∗^*p* < 0.001.

## Data Availability

The data sets for this manuscript will not be publicly available until an associated PhD thesis is published. Requests to access these data sets should be directed to Andra-Diana Andreicut at andra_cecan@yahoo.com.
